# AI EXPLAINABILITY IN LONG-TERM CARE: A SOCIOLOGICAL INQUIRY

**DOI:** 10.1093/geroni/igae098.1115

**Published:** 2024-12-31

**Authors:** Vera Gallistl

**Affiliations:** Karl Landsteiner University of Health Sciences, Krems, Niederosterreich, Austria

## Abstract

Transparency, fairness, and explainability are integral components of contemporary discourses on AI governance, as evidenced by recent policies such as the AI Act by the European Commission. The concept of explainability reflects a growing recognition of the importance to understand AI systems’ operations and decisions, ultimately fostering trust in their utilization (Markus et al., 2021; Ghassemi et al., 2021). While AI explainability is commonly framed within technical parameters, focusing on tools within data science and informatics to elucidate the algorithms behind automated decision-making processes, this perspective tends to overlook the broader, contextual dimensions of AI explainability. In contrast, this paper adopts a sociological lens to explore the multifaceted nature of explainability (Borch & Min 2022) in the context of AI for long-term care settings (LTC). Drawing on insights from multiple-perspective case studies centered on two AI applications—fall detection and social robots— the study investigates how technology developers, care staff, and older adults conceptualize AI explainability. The paper uses data from 30 qualitative interviews and 50 hours of participant observations. The findings highlight the diverse and at times conflicting understandings of explainability that underpin the development and implementation of AI in LTC. They also illustrate how explainability needs of stakeholders in LTC go beyond technological aspects and instead focus on trust, transparency and meaning-making with AI. Finally, the findings underscore the need to broaden concepts around AI explainability to include the more nuanced explainability needs of older adults and avoid ageist tendencies in the AI explainability discourse.

